# Social media use motives and academic performance among Saudi medical students: the mediating role of perceived stress

**DOI:** 10.3389/fmed.2026.1883808

**Published:** 2026-08-17

**Authors:** Ayoob Lone, Imtiyaz Ahmad Dar, Mohammed Farhan AlFarhan, Syed Gulfishan, Abdul Wahab Pathath, Sayed Abdul Qadar Quadri, Nawaf Al Khashram, Abdullah Abdulaziz Alnaim, Maryam Jan

**Affiliations:** 1 Department of Clinical Neurosciences, College of Medicine, King Faisal University, Al Ahsa, Saudi Arabia; 2 Department of Psychology, University of Kashmir, Srinagar, Jammu and Kashmir, India; 3 Department of Surgery, College of Medicine, King Faisal University, Al Ahsa, Saudi Arabia; 4 Department of Biomedical Sciences, College of Medicine, King Faisal University, Al Ahsa, Saudi Arabia; 5 Medical Education Department, College of Medicine, King Faisal University, Al Ahsa, Saudi Arabia; 6 Department of Family and Community Medicine, College of Medicine, King Faisal University, Al Ahsa, Saudi Arabia; 7 Department of School Education, Srinagar, Jammu and Kashmir, India

**Keywords:** academic performance, medical students, perceived stress, Saudi Arabia, social media motives

## Abstract

**Background:**

A growing body of research has documented associations between social media use and perceived stress. However, the psychological mechanisms underlying these associations, particularly the role of perceived stress in the relationship between social media use motives and academic performance among medical students, remain largely unexplored.

**Objective:**

This study examined the relationships between social media use motives and academic performance among Saudi medical students and investigated whether perceived stress (perceived helplessness and lack of self-efficacy) mediates these relationships.

**Methods:**

A cross-sectional study was conducted among Saudi medical students (N = 323). Data were collected using validated self-report measures assessing social media use motives (academic, entertainment, informational, and socialization), perceived stress dimensions, and academic performance (GPA). Parallel mediation analyses were performed using Hayes’ PROCESS macro for SPSS (Model 4) with 5,000 Bootstrap samples. Adjusted models controlled for demographic characteristics, social media use intensity, and the remaining social media use motives.

**Results:**

Academic motives were positively associated with GPA, whereas entertainment and socialization motives were negatively correlated with GPA. Informational motives showed no significant relationship with academic performance. Academic motives were associated with lower perceived stress, whereas socialization motives were associated with higher perceived stress. Entertainment motives were associated with higher perceived stress only in the unadjusted analyses. Perceived helplessness and lack of self-efficacy were both negatively associated with GPA. Mediation analyses demonstrated significant indirect effects of academic motives through lower levels of perceived stress and of socialization motives through increased perceived stress. In contrast, entertainment motives did not demonstrate significant indirect effects after covariate adjustment, and informational motives showed no significant indirect effects.

**Conclusion:**

Social media use motives were differentially associated with academic performance among medical students. Perceived stress partially mediated the associations of academic and socialization motives with GPA, whereas entertainment motives primarily exhibited direct associations with academic performance after adjustment for covariates. These findings highlight the importance of considering both motivational and psychological factors when examining the academic consequences of social media use and underscore the need to promote adaptive social media use and stress-management strategies in medical education.

## Introduction

The rapid expansion of social networking sites (SNSs) has fundamentally transformed how university students communicate, access information, and engage with educational environments (1). Among higher education populations, social media platforms are no longer used solely for interpersonal communication and entertainment; rather, they have become deeply integrated into students’ academic, professional, and social routines (2). Originally developed to facilitate online social interaction, SNSs have evolved into dynamic digital ecosystems that support communication, collaboration, information exchange, content creation, and professional networking (3, 4). Unlike traditional media environments, social media platforms encourage active participation and user-generated content, thereby creating opportunities for collaborative learning, knowledge sharing, and continuous academic interaction (5).

Within higher education contexts, SNSs are increasingly recognized as influential tools capable of shaping students’ learning behaviors, academic engagement, and educational experiences (6–9). Prior research suggests that social media may support academic learning by facilitating peer collaboration, improving access to educational resources, enhancing communication among students, and strengthening engagement in learning activities (10–12). At the same time, growing concerns have emerged regarding the potential psychological and academic consequences of excessive or maladaptive social media engagement. Several studies have linked problematic or non-purposeful SNS use with distraction, procrastination, emotional exhaustion, disrupted study routines, reduced attention span, and poorer academic productivity (13–16).

Despite increasing scholarly attention, findings regarding the relationship between social media use and academic performance remain inconsistent. While some studies report positive associations between SNS engagement and academic outcomes, others identify negative or non-significant relationships (17, 18). These contradictory findings may partly reflect conceptual and methodological limitations within the existing literature. In particular, much of the prior research has conceptualized social media use as a unidimensional construct measured primarily through frequency or duration of use while overlooking the motivations underlying engagement (19, 20). Social media use motives refer to the underlying psychological and functional reasons that drive individuals’ engagement with SNS platforms. Emerging evidence increasingly suggests that the educational and psychological implications of social media use may depend less on the amount of engagement itself and more on the motivations and gratifications driving students’ participation (21, 22). Consequently, understanding why students engage with social media may provide greater explanatory value than examining usage frequency alone.

These issues are particularly important within medical education, where students operate under sustained academic pressure, intensive workloads, time constraints, and high performance expectations (23). Medical training requires substantial cognitive engagement, effective time management, disciplined self-regulation, and continuous academic persistence (24, 25). Simultaneously, SNSs have become increasingly integrated into medical students’ academic routines, frequently functioning as platforms for resource sharing, collaborative discussion, peer interaction, and examination preparation (26). Although these functions may support collaborative learning and academic communication, excessive or poorly regulated engagement may interfere with concentration, reduce productive study time, and impair academic efficiency (27, 28).

Medical students are also particularly vulnerable to elevated levels of stress because of the demanding nature of their educational environment (29, 30). Previous evidence indicates that medical students consistently report higher levels of psychological distress, anxiety, and burnout compared with the general student population (31–33). Persistent academic pressure and competitive learning environments have been consistently associated with impaired concentration, emotional exhaustion, reduced motivation, and poorer academic functioning (34, 35). Within this context, social media engagement may function both as a coping resource and as a potential source of psychological strain. Although students may use SNSs to regulate stress and seek social support, entertainment-oriented or excessive engagement may contribute to heightened stress through mechanisms such as social comparison, information overload, fear of missing out, and disrupted sleep patterns (36–39).

Saudi Arabia provides a particularly important context for examining these relationships because of the exceptionally high prevalence of social media use among young adults and university students. Recent national estimates indicate that by October 2025 the country had approximately 38.6 million social media users, representing one of the highest rates of digital engagement globally (40). Participation rates are especially high among individuals aged 18–24 years, including university students who constitute a substantial proportion of the digitally active population (41, 42). Given the extensive integration of SNSs into students’ academic and social lives, understanding the educational and psychological implications of social media engagement within this population has become increasingly important (43).

Despite growing scholarly interest in social media use among university students, several important gaps remain within the existing literature. First, previous studies examining the relationship between social media engagement and academic performance have produced contradictory findings, with some studies reporting positive associations, others identifying negative relationships, and several demonstrating non-significant effects (16, 17, 42). Second, much of the existing literature has focused primarily on general social media use rather than the underlying motivations driving engagement, despite growing evidence suggesting that motive-specific patterns of use may produce substantially different academic and psychological outcomes (19, 20). Third, although social media engagement has been linked to psychological outcomes such as stress, anxiety, and emotional exhaustion, relatively limited research has examined the psychological mechanisms through which social media use motives are associated with academic performance, particularly among medical students (21, 36). Finally, empirical research examining these relationships within Saudi and broader Gulf educational contexts remains relatively limited despite the exceptionally high prevalence of SNS engagement among university students in the region.

To address these gaps, the present study adopts a multidimensional and theory-driven approach to examine how different social media use motives relate to academic performance among Saudi medical students. More specifically, the study investigates the mediating role of perceived stress in the relationship between social media use motives and academic performance. By focusing on the motivations underlying SNS engagement rather than usage frequency alone, the study seeks to advance current understanding of the psychological and academic implications of social media use within medical education and contribute to theory-driven research on digital behavior among university students.

## Theoretical framework

### Uses and gratifications theory

The present study is primarily grounded in Uses and Gratifications Theory (UGT), which proposes that individuals actively engage with media platforms to satisfy distinct psychological, informational, and social needs (44). Within contemporary digital environments, UGT suggests that users selectively engage with social media platforms based on the gratifications they seek, including entertainment, information acquisition, social interaction, emotional regulation, and task-oriented purposes (19). From this perspective, social media engagement is inherently motive-driven rather than behaviourally uniform.

Accordingly, students may use SNSs for academically oriented purposes such as exchanging educational materials, coordinating coursework, discussing lecture content, and preparing for examinations. Conversely, engagement may also be motivated by entertainment, socialization, avoidance, emotional regulation, or general information-seeking. UGT therefore provides an appropriate theoretical lens for understanding how different social media use motives may produce distinct academic and psychological outcomes among university students.

Importantly, UGT suggests that the consequences of media use depend not merely on the frequency of engagement but on the underlying motivations driving such engagement (22). Consequently, academically oriented motives may facilitate adaptive educational outcomes, whereas entertainment-oriented or avoidance-related motives may contribute to maladaptive academic and psychological consequences.

### Stress and coping perspective

The present study additionally draws upon stress and coping perspectives, particularly the Transactional Model of Stress and Coping, which conceptualizes stress as arising when individuals perceive environmental demands as exceeding their available coping resources (45). Within academic environments, prolonged stress has consistently been associated with poorer concentration, emotional exhaustion, lower motivation, cognitive fatigue, and poorer educational outcomes (34, 46).

From this perspective, perceived stress may represent an important psychological mechanism linking social media use motives with academic performance. Different forms of social media engagement may either alleviate or intensify stress depending on the motivations underlying use and the extent to which engagement supports or disrupts adaptive coping processes. Academically oriented engagement may lower stress by strengthening informational support, collaborative learning, and perceptions of academic preparedness. In contrast, entertainment-oriented, emotionally driven, or avoidance-based engagement may contribute to heightened stress through mechanisms such as cognitive overload, ineffective coping, information overload, and disrupted academic routines (21, 36).

The present study integrates these complementary theoretical perspectives to explain both the motivational antecedents and the psychological mechanisms underlying social media use. Uses and Gratifications Theory explains why students engage with social media for different purposes, whereas the Transactional Model of Stress and Coping explains how these distinct motivations may be associated with the appraisal of academic demands and available coping resources, reflected in perceived helplessness and lack of self-efficacy. Together, these frameworks provide the conceptual basis for proposing that perceived stress serves as the psychological pathway linking social media use motives with academic performance.

**Figure 1:**
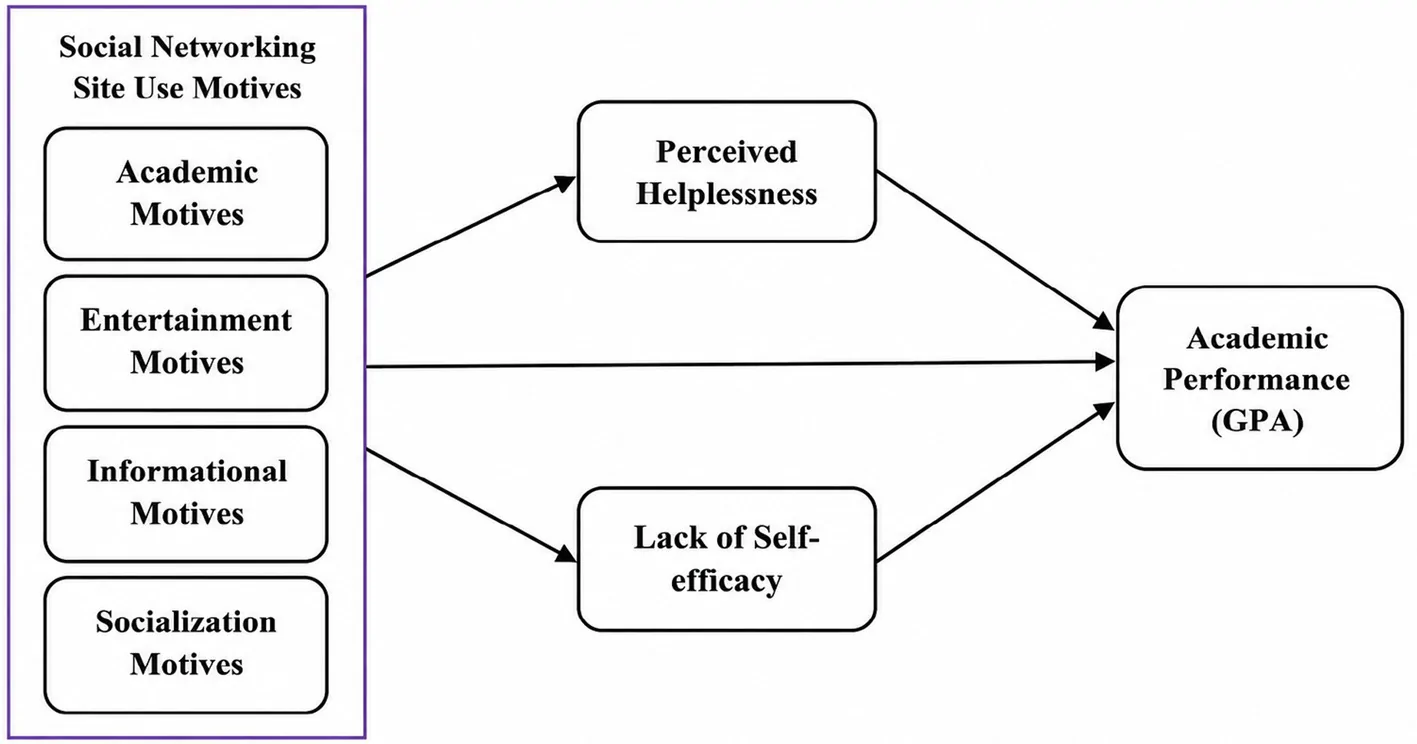
Proposed mediation model of the relationship between social media use motives and academic performance through perceived stress.

Based on the preceding theoretical perspectives and empirical literature, the following hypotheses are proposed. The proposed conceptual framework illustrating the hypothesized relationships among social media use motives, perceived stress, and academic performance is presented in Figure 1.

## Conceptual framework and hypotheses development

### Social media use motives and academic performance

The relationship between social media use and academic performance appears to depend substantially on the motivations underlying engagement. Taken together, prior evidence indicates that academically oriented engagement may facilitate knowledge acquisition, collaborative learning, educational communication, and academic engagement, thereby supporting academic functioning (10, 47, 48). Conversely, entertainment-oriented and socially driven engagement have frequently been associated with distraction, lower study efficiency, procrastination, diminished self-regulation, and poorer academic productivity (13, 27, 28).

From a Uses and Gratifications Theory perspective, these inconsistencies may reflect differences in the underlying motives driving SNS engagement rather than social media use per se. Because users engage with social media to satisfy distinct psychological and functional needs, the academic implications of SNS use are likely to vary according to the specific gratifications being sought through engagement (44).

*H1*: Social media use motives are significantly associated with academic performance among Saudi medical students.

### Social media use motives and perceived stress

Perceived stress refers to the extent to which individuals appraise life circumstances as overwhelming, unpredictable, or exceeding available coping resources (49). Within university settings, elevated stress has consistently been associated with impaired cognitive functioning, emotional exhaustion, lower concentration, and poorer psychological well-being (34, 50).

Emerging evidence suggests that social media engagement may differentially relate to stress depending on the motivations underlying use. From a stress and coping perspective, students may engage with social media to seek emotional support, regulate emotions, or temporarily escape academic pressure (38, 51). However, not all forms of engagement appear equally adaptive. Entertainment-oriented, socially driven, or avoidance-related engagement may contribute to heightened stress through mechanisms such as upward social comparison, fear of missing out, information overload, disrupted sleep patterns, and ineffective time management (36, 37, 39).

Moreover, passive or emotionally driven forms of SNS engagement appear more strongly associated with stress-related outcomes than active and purposeful engagement (52). These dynamics may be particularly relevant among medical students because of the sustained academic demands associated with medical training (53, 54).

*H2*: Social media use motives are significantly associated with perceived stress among Saudi medical students.

### Perceived stress and academic performance

Perceived stress has emerged as a major psychological factor associated with students’ emotional well-being, cognitive functioning, and academic outcomes. Medical students are particularly vulnerable because of intensive coursework, frequent examinations, high expectations, and continuous academic competition (55, 56).

A substantial body of evidence suggests that elevated stress is associated with poorer academic functioning and lower educational performance among university students (50). Excessive stress may impair concentration, memory, executive functioning, motivation, and self-regulatory capacity, thereby negatively affecting academic engagement and learning efficiency (46, 57).

According to the Transactional Model of Stress and Coping, prolonged exposure to stress may exceed students’ coping resources, resulting in emotional exhaustion and impaired academic functioning (45). Within medical education contexts, persistent stress may therefore undermine students’ ability to effectively manage academic demands and maintain educational performance.

*H3*: Perceived stress is significantly associated with academic performance among Saudi medical students.

### Mediating role of perceived stress

Although social media use motives may demonstrate direct associations with academic performance, their relationships with academic functioning may also operate through underlying psychological mechanisms. Drawing upon Uses and Gratifications Theory alongside stress-based perspectives, perceived stress may represent an important explanatory pathway linking social media engagement motives with academic outcomes.

More specifically, entertainment-oriented, socially driven, or avoidance-related engagement may contribute to elevated stress perceptions through mechanisms such as cognitive overload, emotional exhaustion, ineffective coping, and disrupted academic routines (36, 37). Elevated stress may subsequently interfere with concentration, motivation, academic organization, and effective self-regulation, thereby impairing academic performance (46, 57). Conversely, academically oriented engagement may be associated with lower stress because it often involves collaborative learning, greater access to educational resources, and stronger perceptions of academic preparedness and control (47, 48).

Accordingly, the effects of social media use motives on academic performance may occur indirectly through students’ stress perceptions and coping responses. Recent evidence increasingly supports the role of stress-related psychological mechanisms in explaining associations between social media engagement and academic functioning (21, 58). Thus, perceived stress may help explain how different social media use motives are associated with either adaptive or maladaptive academic outcomes among medical students.

*H4*: Perceived stress significantly mediates the relationship between social media use motives and academic performance among Saudi medical students.

### The present study

Building upon Uses and Gratifications Theory and stress-based perspectives, the present study adopts a multidimensional approach to examine how distinct social media use motives relate to academic performance among Saudi medical students. More specifically, the study investigates the mediating role of perceived stress in the relationship between social media use motives and academic performance. By focusing on motive-specific engagement rather than usage frequency alone, the study seeks to advance current understanding of the psychological and academic implications of social media use within medical education and contribute to theory-driven research on digital behavior among university students.

## Methods and materials

### Study design and participants

A cross-sectional study was conducted between November 2025 and February 2026 among undergraduate medical students enrolled at the College of Medicine, King Faisal University. Since the students represent various geographical regions of Saudi Arabia, the participant population shows geographical diversity, even though they are from the same institution. The study participants were chosen using a convenient sampling method through the on-site distribution of the study questionnaire. The Deanship of Scientific Research at King Faisal University granted ethical approval (KFU-REC-2025-AUG–ETHICS3463). All study procedures adhered to the ethical guidelines of the Helsinki Declaration and relevant guidelines for ethical research involving human subjects (59). Participants were informed about the study’s objectives, how responses would remain confidential, that the study was voluntary, and that they could withdraw at any time without consequence. All participants provided electronic informed consent prior to participation. The minimum required sample size, using Yamane’s (60) formula for finite populations with a 5% margin of error (61), was 322 participants. In order to enhance response adequacy and compensate for prospective incomplete responses, 400 students were invited. Of the 400 students, 339 accepted the invitation with a response rate of 84.8%. Prior to the analysis, sixteen of the returned questionnaires were excluded due to a significant number of missing responses. The final analysis included 323 survey respondents.

### Instruments

To achieve the study’s objectives, a structured questionnaire was created with three primary sections. The first section consists of socio-demographic aspects of the participants, including sex, age, academic year, social media accounts, as well as the monthly financial earnings of their families. Additionally, participants were asked to report their academic performance from the previous semester using a standardized GPA classification system. To preserve confidentiality and avoid requesting exact GPA values, academic performance was assessed using a five-point ordinal GPA classification system ranging from 1 (GPA 4.5–5.0) to 5 (GPA below 2.0). To facilitate interpretation, GPA categories were reverse-coded such that higher values reflected better academic performance. Instead of asking students to report their exact GPA, we used predefined ranges, as students may not always recall precise scores or may feel uncomfortable reporting them. Using categories also helped lower response burden and ensured that most participants were able to provide this information. For the analyses, each category was represented by its midpoint (for example, 4.75 for the 4.5–5.0 range), which allowed GPA to be treated as approximately continuous in the regression models. This step made the analyses more straightforward, although it should be noted that it does not capture the full precision of actual GPA values. To check that our findings were not dependent on this decision, we also ran ordinal regression models using GPA in its original categorical form, and the overall pattern of results remained similar. Participants were also asked to provide their estimates regarding social media time. This item was based on the previous study (62), which used the seven-point scale to measure social media time, with 1 as one or less hour per day to 7 as seven or more hours a day. The second phase of the study analyzed social media use motives through the Social Networking Usage Questionnaire (SNUQ) developed by Gupta and Bashir (63). This instrument consists of 19 questions divided into four categories: academic use (7 questions), social use (5 questions), entertainment (4 questions), and information gathering (3 questions). The five-point Likert scale indicates the user’s level of social networking engagement with 1 (never) and 5 (always). Therefore, higher scores signal higher engagement in social networking. Although the SNUQ was originally developed to assess patterns of social networking use, its four dimensions closely correspond to gratification-oriented forms of social media engagement described within the Uses and Gratifications framework. Consequently, several previous studies have operationalized these dimensions as indicators of social media use motives rather than merely frequency of use [e.g., (62, 64)]. Accordingly, the present study employed the four SNUQ dimensions as measures of academic, socialization, entertainment, and informational motives for social media use. To further evaluate the appropriateness of this operationalization in the present sample, discriminant validity was examined using the Fornell–Larcker criterion and the heterotrait–monotrait ratio (HTMT). Results demonstrated adequate discriminant validity for all four constructs. Notably, the academically and conceptually related academic and informational motive dimensions satisfied both criteria, confirming that they represented empirically distinct constructs despite their conceptual overlap. These findings provide additional evidence supporting the use of the SNUQ dimensions as separate measures of social media use motives in the present study. Furthermore, the SNUQ has demonstrated satisfactory psychometric properties across different cultural settings, including studies conducted in Arab populations (65, 66). The original validation study reported good internal consistency (Cronbach’s *α* = 0.83). Section three of the questionnaire measured the perceived stress scale (PSS) as designed by Cohen et al. (49). The scale comprised 10 questions, which had ratings based on a five-point Likert scale. These included options from ‘never’ (0) to ‘very often’ (4), with questions 4, 5, 7, and 8 being reverse-scored. The perceived stress score for each individual was the sum of all the responses. The score ranges 0–13, 14–26, and 27–40 are considered low, moderate, and high perceived stress, respectively. The scale showed a high degree of internal consistency reliability at Cronbach’s alpha = 0.80. Although the PSS-10 is commonly used as a unidimensional measure of perceived stress, substantial psychometric evidence supports a two-factor structure comprising perceived helplessness and perceived self-efficacy across diverse cultural and student populations, including Arab samples (67–69). Consistent with this literature and the objectives of the present study, perceived stress was operationalized through these two dimensions. To establish construct validity within the present sample, a confirmatory factor analysis (CFA) was conducted to evaluate the two-factor structure of the PSS-10. The model demonstrated acceptable fit to the data, χ^2^(34) = 123.00, *p* < 0.001, CFI = 0.928, TLI = 0.905, SRMR = 0.050, and RMSEA = 0.090 (90% CI [0.073, 0.107]). All standardized factor loadings were statistically significant (*p* < 0.001) and ranged from 0.59 to 0.79. Furthermore, the two latent factors were positively correlated (*r* = 0.17, *p* = 0.010), supporting their conceptual relatedness while indicating empirical distinctiveness. The reliability coefficients of data collection tools can be found in Table 1.

**Table 1:** Descriptive statistics and reliability indices for study scales (*N* = 323).

Scales	No. of Items	Mean	SD	Skewness	Kurtosis	Cronbach’s α
Social media motives						
Academic Motives	7	22.59	6.66	−0.21	−0.95	0.809
Socialization Motives	5	15.68	4.61	0.11	−0.72	0.820
Entertainment Motives	4	14.12	3.05	0.10	−0.62	0.783
Informational Motives	3	8.87	3.08	0.16	−0.90	0.737
Perceived stress						
Perceived Helplessness	6	13.81	5.11	−0.28	−0.50	0.877
Lack of Self-Efficacy	4	9.41	2.88	0.12	−0.49	0.760

### Procedure

After obtaining institutional approval, recruitment was conducted through student representatives across academic years. Survey information and participation links were disseminated through academic WhatsApp groups following permission from group administrators. Prior to data collection, a pilot study involving 30 students was conducted to assess the clarity and comprehensibility of the questionnaire. Data obtained from the pilot study were not included in the final analyses. Interested participants were contacted by trained senior medical students who provided information regarding the study procedures before participation.

### Statistical analysis

All analyses were conducted using IBM SPSS Statistics (Version 27). Prior to hypothesis testing, the dataset was screened to ensure accuracy and suitability for statistical analyses. All responses were examined for data entry errors, and no inconsistencies were identified. During data screening, 16 questionnaires containing substantial missing responses were excluded from the study. Consequently, the final analytical sample consisted only of participants who provided complete responses on all study variables, and no missing data were present in the dataset used for statistical analyses. The data were screened for outliers. Univariate outliers were assessed using standardized z-scores, with values exceeding ±3.29 considered extreme at the *p* < 0.001 level (70, 71). No cases met this criterion. Multivariate outliers were examined using Mahalanobis distance. Based on the chi-square criterion for 11 variables at *p* < 0.001, no cases exceeded the critical threshold (70). The maximum observed Mahalanobis distance (D^2^ = 26.07, *p* = 0.006) remained below the critical chi-square threshold, indicating that no multivariate outliers were present and all cases were retained for analysis.

Normality was evaluated by inspecting skewness and kurtosis values for all continuous study variables. Following commonly cited guidelines (71–73), skewness values within ±3 and kurtosis values within ±10 were considered acceptable. In the present dataset, skewness values ranged from −0.82 to 0.19, while kurtosis values ranged from −1.18 to −0.05, indicating that the variables were approximately normally distributed. Multicollinearity was assessed using tolerance and variance inflation factor (VIF) values (70, 72, 74). Although most predictors fell within acceptable limits, age and academic year demonstrated elevated VIF values, indicating substantial shared variance. Given this overlap, age was excluded from subsequent analyses, and academic year was retained as the more theoretically meaningful indicator of academic progression. All remaining predictors showed acceptable tolerance and VIF values, indicating that multicollinearity was not a concern. Common method bias was assessed using Harman’s single-factor test (75). The first unrotated factor accounted for less than 30% of the total variance, suggesting that common method bias was unlikely to pose a serious threat to the validity of the findings. In addition to this statistical test, several procedural remedies were implemented to minimize common method bias, including assuring participants of anonymity and confidentiality to lower evaluation apprehension, and employing validated instruments with conceptually distinct constructs.

Hypotheses were tested using Hayes’ PROCESS macro [Model 4; (76)]. Parallel mediation analyses were conducted using Model 4 to examine whether perceived stress dimensions (lack of self-efficacy and perceived helplessness) mediated the relationships between social media motives and academic performance (GPA). Separate mediation models were estimated for each social media use motive dimension. In each model, one motive served as the focal independent variable, while the remaining three motive dimensions were entered as covariates together with demographic and social media use variables. Thus, all mediation models estimated the unique association of each motive after adjustment for shared variance with the other motives. Indirect effects were estimated using 5,000 bootstrap resamples to generate bias-corrected 95% confidence intervals. Effects were considered statistically significant when the corresponding confidence interval did not include zero. This bootstrapping approach is recommended for mediation analysis because it provides more accurate estimation of indirect effects and better control of Type I error compared to normal-theory methods (76, 77).

## Results

### Bivariate correlations

Bivariate correlations among all study variables are presented in Table 2. Several demographic variables, including academic year, family income, and number of social media accounts, showed significant associations with the independent, dependent, and mediating variables. These findings suggest that demographic and usage-related characteristics were meaningfully related to key study constructs at the zero-order level.

**Table 2:** Pearson correlations and multicollinearity diagnostics (*N* = 323).

Variable	1	2	3	4	5	6	7	8	9	10	11	12	Tolerance	VIF
1. GPA	—												—	—
2. Socialization Motives	−0.40***	—											0.70	1.44
3. Academic motives	0.65***	−0.16***	—										0.61	1.64
4. Informational motives	0.05	0.22***	0.26***	—									0.83	1.21
5. Entertainment motives	−0.47***	0.35***	−0.41***	0.03	—								0.71	1.41
6. Time spent	−0.21***	0.19***	−0.12**	0.07	0.10	—							0.89	1.13
7. Lack of Self-Efficacy	−0.47***	0.27***	−0.33***	−0.08	0.22***	0.10	—						0.83	1.21
8. Perceived Helplessness	−0.49***	0.31***	−0.40***	0.02	0.27***	0.16***	0.15***	—					0.77	1.31
9. Age	−0.05	0.01	0.03	0.14**	−0.05	0.05	0.02	−0.05	—				0.10	9.79
10. Academic year	−0.02	−0.03	0.07	0.16***	−0.03	0.07	0.00	−0.07	0.94***	—			0.10	9.70
11. Family income	0.18***	−0.07	0.09	−0.01	−0.06	0.00	−0.07	−0.03	0.13**	−0.08	—		0.82	1.22
12. No. of SM accounts	0.10	−0.13**	0.06	0.02	0.08	0.16***	−0.12**	0.01	−0.21***	−0.14**	0.40***	—	0.73	1.38

**p < 0.05, ***p < 0.01.

Because age and academic year were highly correlated (*r* = 0.94, VIF > 9), only academic year was retained in the adjusted mediation analyses to reduce multicollinearity concerns. Sex was also included as a control variable based on prior evidence demonstrating differences in social media use and academic outcomes among students (78). Family income and number of social media accounts were retained as additional covariates because of their observed associations with study variables.

Although academic and informational motives were positively correlated, the magnitude of the association remained within acceptable limits and did not indicate problematic multicollinearity, thereby supporting the discriminant validity of the study variables and justifying their inclusion in subsequent mediation analyses. Overall, the correlation patterns provided preliminary support for the proposed relationships and justified the inclusion of covariates in subsequent mediation analyses.

Importantly, the tolerance and VIF values reported in Table 2 correspond to the predictors included in the adjusted mediation models. All retained predictors demonstrated acceptable tolerance values (> 0.60) and VIF values (< 2.00), indicating that multicollinearity was unlikely to have materially influenced the regression estimates. Accordingly, the adjusted mediation models showed no evidence of problematic multicollinearity, supporting the stability and interpretability of the estimated regression coefficients.

### Distributional characteristics and reliability of the study instruments

As presented in Table 1, the study scales demonstrated satisfactory psychometric properties within the present sample. Mean scores ranged from 8.87 to 22.59, indicating adequate variability across constructs, while standard deviations suggested sufficient dispersion in participant responses. Skewness values ranged from −0.28 to 0.16, and kurtosis values ranged from −0.95 to −0.49, indicating no substantial deviations from normality. Internal consistency estimates were acceptable to good across all measures, with Cronbach’s alpha coefficients ranging from 0.74 to 0.88. Perceived helplessness demonstrated the highest reliability, whereas informational motives showed comparatively lower, yet acceptable, reliability. Taken together, these findings support the reliability and psychometric adequacy of the measures used in the present study.

### Mediation analyses

**Figure 2:**
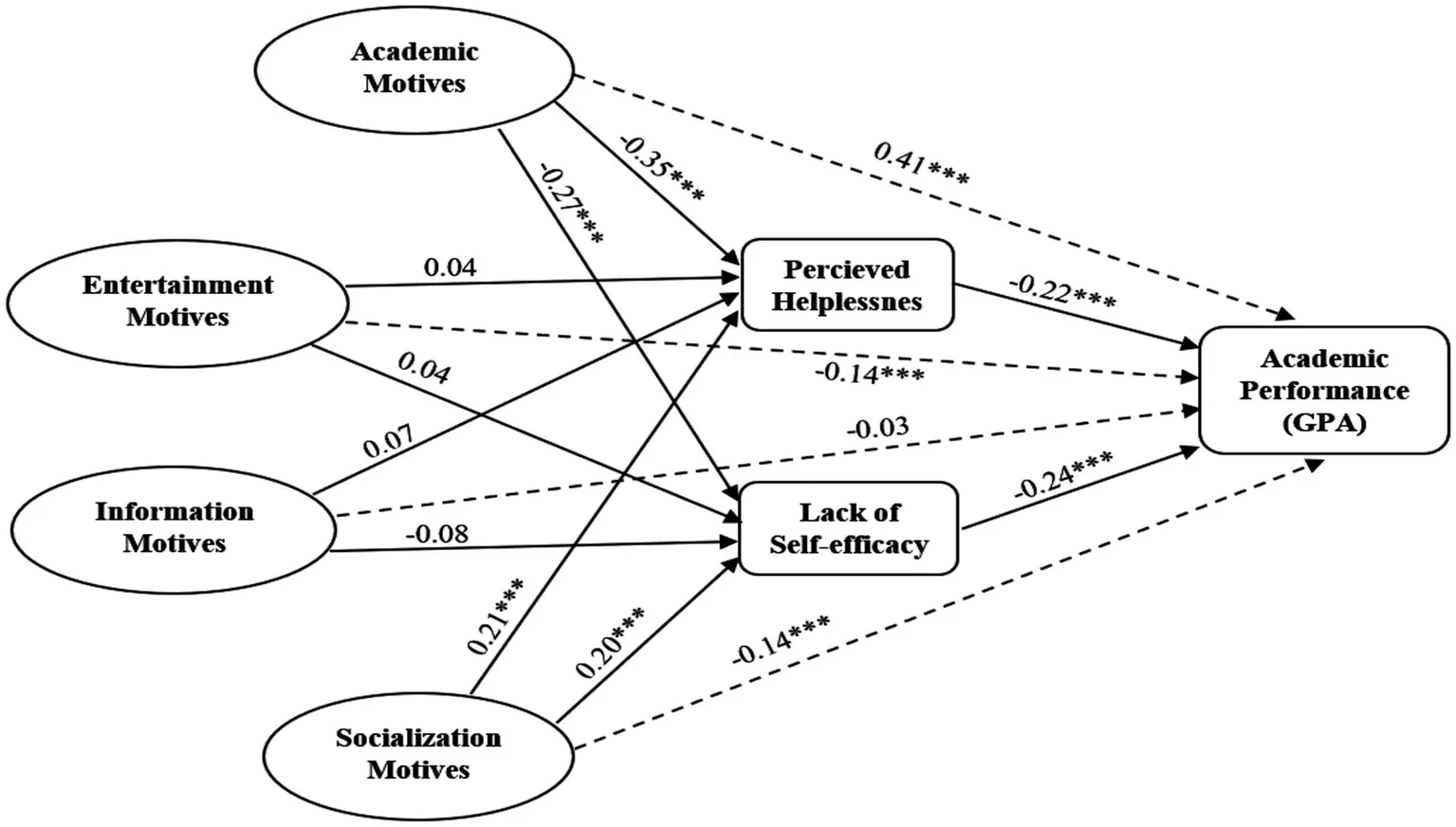
Parallel mediation model showing the associations between social media use motives, perceived stress dimensions, and academic performance. Standardized path coefficients for the adjusted model are presented. Solid arrows represent indirect pathways through the mediators, whereas dotted arrows represent direct pathways to academic performance (GPA). ***p < 0.001.

Parallel mediation analyses were performed using Hayes’ PROCESS macro for SPSS [Model 4; (76)] with 5,000 bias-corrected bootstrap resamples to examine whether two dimensions of perceived stress— perceived helplessness and lack of self-efficacy—were associated with the relationships between social media motives (academic, entertainment, informational, and socialization motives) and academic performance (GPA). Unadjusted models were estimated first, followed by adjusted models controlling for demographic characteristics, social media use indicators, and the remaining social media use motives. Regression coefficients are presented in Table 3, and bootstrapped indirect effects are reported in Table 4. The adjusted parallel mediation model illustrating the direct and indirect relationships among the study variables is presented in Figure 2.

**Table 3:** Parallel mediation analysis (PROCESS Model 4; *N* = 323).

	Unadjusted model				Adjusted model			
	*B*	*β*	SE	*t*	*B*	*β*	SE	*t*
Mediator model (X → M1: LSE)								
Academic motives	−0.144***	−0.333	0.023	−6.33	−0.118***	−0.272	0.027	−4.37
Entertainment motives	0.209***	0.221	0.051	4.07	0.041	0.044	0.057	0.72
Informational motives	−0.078	−0.083	0.052	−1.50	−0.076	−0.081	0.053	−1.42
Socialization motives	0.171***	0.273	0.034	5.09	0.128***	0.205	0.037	3.42
Mediator model (X → M2: PH)								
Academic motives	−0.306***	−0.398	0.039	−7.77	−0.268***	−0.349	0.046	−5.83
Entertainment motives	0.460***	0.274	0.090	5.10	0.071	0.042	0.098	0.73
Informational motives	0.031	0.019	0.093	0.34	0.111	0.067	0.091	1.22
Socialization motives	0.338***	0.305	0.059	5.73	0.238***	0.215	0.064	3.73
Outcome model (M → Y)								
LSE → GPA	−0.058***	−0.293	0.008	−7.66	−0.047***	−0.237	0.007	−6.43
PH → GPA	−0.032***	−0.287	0.004	−7.30	−0.025***	−0.222	0.004	−5.79
Total effects (c)								
Academic motives → GPA	0.057***	0.661	0.004	15.76	0.047***	0.548	0.004	12.20
Entertainment motives → GPA	−0.089***	−0.473	0.009	−9.62	−0.030***	−0.158	0.008	−3.62
Informational motives → GPA	0.009	0.047	0.010	0.85	−0.005	−0.025	0.008	−0.60
Socialization motives → GPA	−0.050***	−0.404	0.006	−7.91	−0.029***	−0.232	0.005	−5.37
Direct effects (c′)								
Academic motives → GPA	0.039***	0.449	0.004	10.89	0.035***	0.406	0.004	9.23
Entertainment motives → GPA	−0.054***	−0.290	0.008	−6.95	−0.026***	−0.139	0.007	−3.50
Informational motives → GPA	0.004	0.021	0.008	0.49	−0.005	−0.029	0.007	−0.78
Socialization motives → GPA	−0.022***	−0.180	0.006	−4.03	−0.017***	−0.136	0.005	−3.34

*B*, unstandardized coefficient; *β*, standardized coefficient; *SE*, standard error; LSE, Lack of Self-efficacy; PH, Perceived Helplessness. ***p < 0.001. Adjusted models controlled for academic year, sex, family income, number of social media accounts, time spent on social networking sites, and the remaining social media use motive dimensions (academic, entertainment, informational, or socialization motives, as applicable). For each model, the focal motive was entered as the predictor while the other three motive dimensions were included as covariates.

**Table 4:** Indirect effects (bootstrapped; PROCESS Model 4, *N* = 323).

Predictor	Path	Unadjusted model				Adjusted model			
		*B*	Boot SE	95% CI	*β*(cs)	*B*	Boot SE	95% CI	*β*(cs)
Academic motives	via LSE	0.0084	0.0018	[0.0051, 0.0121]	0.0977	0.0055	0.0017	[0.0026, 0.0091]	0.0645
	via PH	0.0098	0.0017	[0.0066, 0.0134]	0.1141	0.0066	0.0016	[0.0037, 0.0101]	0.0774
	Total	0.0182	0.0029	[0.0129, 0.0240]	0.2118	0.0122	0.0026	[0.0075, 0.0177]	0.1419
Entertainment motives	via LSE	−0.0151	0.0042	[−0.0242, −0.0077]	−0.0808	−0.0019	0.0028	[−0.0077, 0.0036]	−0.0103
	via PH	−0.0192	0.0043	[−0.0283, −0.0114]	−0.1027	−0.0018	0.0024	[−0.0068, 0.0027]	−0.0094
	Total	−0.0344	0.0071	[−0.0494, −0.0211]	−0.1835	−0.0037	0.0041	[−0.0122, 0.0043]	−0.0197
Informational motives	via LSE	0.0064	0.0046	[−0.0026, 0.0153]	0.0347	0.0036	0.0028	[−0.0018, 0.0092]	0.0192
	via PH	−0.0016	0.0047	[−0.0113, 0.0077]	−0.0084	−0.0028	0.0023	[−0.0076, 0.0015]	−0.0149
	Total	0.0049	0.0073	[−0.0098, 0.0187]	0.0263	0.0008	0.0035	[−0.0062, 0.0075]	0.0044
Socialization motives	via LSE	−0.0128	0.0029	[−0.0189, −0.0076]	−0.1030	−0.0060	0.0020	[−0.0101, −0.0024]	−0.0486
	via PH	−0.0150	0.0029	[−0.0210, −0.0096]	−0.1212	−0.0059	0.0018	[−0.0098, −0.0028]	−0.0477
	Total	−0.0278	0.0044	[−0.0366, −0.0196]	−0.2242	−0.0119	0.0026	[−0.0174, −0.0070]	−0.0963

B, unstandardized indirect effect; Boot SE, bootstrap standard error (5,000 samples); CI, bias-corrected 95% confidence interval; β(cs), completely standardized indirect effect, interpreted as an indicator of the magnitude of the mediation pathway. Indirect effects are statistically significant when the confidence interval does not include zero; LSE, Lack of self-efficacy; PH, Perceived helplessness.

Across predictors, the full outcome models predicting GPA were statistically significant in the unadjusted analyses, *F*(3, 319) = 80.71–149.98, *p* < 0.001, explaining approximately 43.2 to 58.5% of the variance in GPA (R^2^ = 0.432–0.585). The corresponding total-effect models were also statistically significant for academic, entertainment, and socialization motives, *F*(1, 321) = 62.53–248.38, *p* < 0.001, accounting for 16.3 to 43.6% of the variance in GPA (R^2^ = 0.163–0.436). In contrast, the total-effect model for informational motives was not statistically significant, *F*(1, 321) = 0.72, *p* = 0.398, R^2^ = 0.002. The inclusion of the mediators increased the variance explained in GPA across all unadjusted models, with ΔR^2^ values ranging from 0.149 to 0.429. Following covariate adjustment, the outcome models remained highly significant, *F*(11, 311) = 52.61, *p* < 0.001, explaining 65.1% of the variance in GPA (R^2^ = 0.651). The corresponding total-effect models were also significant, *F*(9, 313) = 45.96, *p* < 0.001, accounting for 56.9% of the variance in GPA (R^2^ = 0.569). Thus, inclusion of the mediators accounted for an additional 8.1% of explained variance in GPA (ΔR^2^ = 0.081) beyond that explained by the social media motive dimensions and covariates. The adjusted mediator models explained 17.5% of the variance in lack of self-efficacy (R^2^ = 0.175) and 23.5% of the variance in perceived helplessness (R^2^ = 0.235). Consistent with the multicollinearity diagnostics presented in Table 2, the adjusted mediation models satisfied conventional assumptions regarding predictor independence, indicating that the observed parameter estimates were not materially affected by multicollinearity. In addition to statistical significance, the magnitude of the observed associations was evaluated using standardized regression coefficients (*β*), completely standardized indirect effects [*β*(cs)], and explained variance (R^2^ and ΔR^2^).

Overall, inclusion of demographic characteristics, social media use indicators, and competing motive dimensions substantially improved model explanatory power. Nevertheless, significant indirect effects in the adjusted analyses were observed only for academic and socialization motives, indicating that mediation was not supported for entertainment or informational motives after controlling for covariates.

Across models, both perceived helplessness and lack of self-efficacy demonstrated consistent negative associations with GPA. In the unadjusted analyses, lack of self-efficacy was negatively associated with GPA (*β* = −0.29 to −0.42, *p* < 0.001), as was perceived helplessness (*β* = −0.29 to −0.45, *p* < 0.001). Following covariate adjustment, both associations remained statistically significant, although somewhat attenuated (lack of self-efficacy: *β* = −0.24, *p* < 0.001; perceived helplessness: *β* = −0.22, *p* < 0.001). These findings indicate that higher levels of perceived stress were associated with poorer academic performance independent of demographic characteristics, social media use indicators, and other social media motives (see Table 3).

### Indirect effects

Bootstrap analyses for the unadjusted models indicated that academic motives demonstrated statistically significant positive indirect effects on GPA through both lack of self-efficacy and perceived helplessness. Specifically, the indirect effect via lack of self-efficacy was *β*(cs) = 0.0977, 95% CI [0.0051, 0.0121], and via perceived helplessness was *β*(cs) = 0.1141, 95% CI [0.0066, 0.0134]. The total indirect effect was also statistically significant (*β*(cs) = 0.2118, 95% CI [0.0129, 0.0240]).

Entertainment motives demonstrated statistically significant negative indirect effects through both mediators. The indirect effect via lack of self-efficacy was *β*(cs) = −0.0808, 95% CI [−0.0242, −0.0077], and via perceived helplessness was *β*(cs) = −0.1027, 95% CI [−0.0283, −0.0114]. The total indirect effect was statistically significant (*β*(cs) = −0.1835, 95% CI [−0.0494, −0.0211]).

Socialization motives also demonstrated statistically significant negative indirect effects through both lack of self-efficacy and perceived helplessness. The indirect effect via lack of self-efficacy was *β*(cs) = −0.1030, 95% CI [−0.0189, −0.0076], and via perceived helplessness was *β*(cs) = −0.1212, 95% CI [−0.0210, −0.0096]. The total indirect effect was statistically significant (*β*(cs) = −0.2242, 95% CI [−0.0366, −0.0196]).

In contrast, informational motives did not exhibit statistically significant indirect effects. The indirect effect via lack of self-efficacy was *β*(cs) = 0.0347, 95% CI [−0.0026, 0.0153], and via perceived helplessness was *β*(cs) = −0.0084, 95% CI [−0.0113, 0.0077]. The total indirect effect was not significant (*β*(cs) = 0.0263, 95% CI [−0.0098, 0.0187]).

Following covariate adjustment, academic motives continued to demonstrate significant indirect effects through lack of self-efficacy (*β*(cs) = 0.0645, 95% CI [0.0320, 0.1033]) and perceived helplessness (*β*(cs) = 0.0774, 95% CI [0.0457, 0.1133]), yielding a significant total indirect effect (*β*(cs) = 0.1419, 95% CI [0.0919, 0.1974]). Similarly, socialization motives retained significant indirect effects through lack of self-efficacy (*β*(cs) = −0.0486, 95% CI [−0.0813, −0.0192]) and perceived helplessness (*β*(cs) = −0.0477, 95% CI [−0.0784, −0.0225]), resulting in a significant total indirect effect (*β*(cs) = −0.0963, 95% CI [−0.1369, −0.0565]).

In contrast, entertainment motives no longer demonstrated significant indirect effects after adjustment for covariates. The total indirect effect was non-significant (*β*(cs) = −0.0197, 95% CI [−0.0651, 0.0232]), and the confidence intervals for both the lack of self-efficacy pathway (*β*(cs) = −0.0103, 95% CI [−0.0405, 0.0192]) and the perceived helplessness pathway (*β*(cs) = −0.0094, 95% CI [−0.0364, 0.0146]) included zero. Informational motives likewise remained non-significant following covariate adjustment.

Overall, after controlling for demographic characteristics, social media use indicators, and competing motive dimensions, significant mediation effects were retained only for academic and socialization motives. Completely standardized indirect effects [*β*(cs)] and their bootstrapped confidence intervals are presented in Table 4. Accordingly, interpretation of the findings was based on both statistical significance and effect size estimates. Standardized regression coefficients (*β*), completely standardized indirect effects [*β*(cs)], and measures of explained variance (R^2^ and ΔR^2^) were considered to evaluate not only whether effects were statistically significant but also their practical magnitude.

### Total and direct effects

Academic motives demonstrated a positive and statistically significant total effect on GPA (*β* = 0.66, *p* < 0.001), which remained significant after inclusion of the mediators (*β* = 0.45, *p* < 0.001), indicating that the association persisted alongside the indirect pathways. Entertainment motives demonstrated a statistically significant negative total effect on GPA (*β* = −0.47, *p* < 0.001). This association remained significant in the direct-effect model (*β* = −0.29, *p* < 0.001), suggesting that the relationship was only partially accounted for by the mediators. Similarly, socialization motives demonstrated a significant negative total effect on GPA (*β* = −0.40, *p* < 0.001), which was attenuated but remained statistically significant after inclusion of the mediators (*β* = −0.18, *p* < 0.001). In contrast, informational motives did not demonstrate a statistically significant total effect (*β* = 0.05, *p* = 0.398) or direct effect (*β* = 0.02, *p* = 0.622), indicating no evidence of an association with GPA in either model.

Following covariate adjustment, the overall pattern of results remained largely unchanged. Academic motives continued to demonstrate significant positive total (*β* = 0.55, *p* < 0.001) and direct effects (*β* = 0.41, *p* < 0.001), whereas entertainment motives retained significant negative total (*β* = −0.16, *p* < 0.001) and direct effects (*β* = −0.14, *p* < 0.001). Socialization motives likewise continued to exhibit significant negative total (*β* = −0.23, *p* < 0.001) and direct effects (*β* = −0.14, *p* = 0.001). In contrast, informational motives remained unrelated to GPA, with both total (*β* = −0.02, *p* = 0.547) and direct effects (*β* = −0.03, *p* = 0.436) remaining non-significant. Although the magnitudes of several effects were attenuated following adjustment, their direction and statistical significance were largely preserved (see Table 3).

### Sensitivity analysis using ordinal GPA

To evaluate the robustness of the findings, supplementary mediation analyses were conducted using the ordinal GPA measure. The pattern of results remained substantively unchanged. Academic motives continued to demonstrate significant positive direct and indirect effects on GPA through both lack of self-efficacy and perceived helplessness, whereas socialization motives retained significant negative direct and indirect effects. In contrast, entertainment motives exhibited significant total and direct effects but non-significant indirect effects, and informational motives remained unrelated to GPA at both the direct and indirect levels. Overall, the consistency of findings across continuous and ordinal operationalisations of academic performance supports the robustness of the observed mediation patterns.

## Discussion

This study investigated the relationships between different motives for social media use and academic performance among Saudi medical students, while also examining whether these relationships operate indirectly through perceived stress, operationalized as perceived helplessness and lack of self-efficacy. Drawing upon Uses and Gratifications Theory (UGT) and Transactional Model of Stress and Coping, the study proposed that social media use is not a uniform behavior but rather a motive-driven process that is associated with psychological stress and academic outcomes. Overall, the findings provide strong evidence for a differentiated, psychologically mediated framework in which perceived stress is associated with the relationship between social media use motives with academic performance. The present findings are consistent with previous studies examining the relationship between social media use motives and GPA (10, 11, 13, 16–18, 36, 62). At the level of direct relationships, the findings showed clear evidence that social media use motives are differentially associated with academic performance, thereby partially supporting H1. Academic motives demonstrated a strong and consistent positive association with GPA, indicating that students who engage with social media for academic purposes tend to perform better academically. These findings are consistent with previous research indicating that academically oriented social media use can positively facilitate academic achievement (11, 21, 79). One possible explanation is that academically motivated social media use may be associated with more purposeful and structured learning behaviors. Students may utilize social media platforms for academic purposes to access educational resources, participate in academic discussions, exchange study materials, and collaborate with peers, all of which may be associated with greater learning engagement and academic productivity. Such goal-directed engagement may additionally be related to stronger self-regulated learning through improved time management, academic planning, and active participation in educational activities. Moreover, academically oriented social media use may be associated with higher perceived academic competence and motivation, thereby potentially contributing to better academic performance.

In contrast, entertainment and socialization motives were consistently negatively associated with GPA, indicating that students who primarily use social media for leisure or social interaction tended to report poorer academic performance. These findings are consistent with previous studies showing that entertainment-oriented social media use is negatively associated with academic achievement (62, 80, 81). A possible explanation is that these motives may be associated with competing demands on students’ time and attention, which may be associated with lower engagement in academic tasks. However, behavioral mechanisms were not directly assessed in this study; therefore, interpretations regarding specific behaviors remain tentative.

Similarly, the negative association between socialization motives and GPA aligns with previous literature reporting that socially driven use of social media may distract students from academic activities and lower academic performance (62, 82). Students who use social media primarily for social interaction may devote substantial time to maintain online communication and manage digital social relationships, potentially interfering with academic focus and study efficiency. Unlike face-to-face communication, online interactions often involve continuous engagement, repetitive checking behaviors, and prolonged conversational exchanges, which have been associated with greater attentional fragmentation and poorer academic concentration. In addition, socialization-oriented use may be associated with greater susceptibility to social comparison and perceived pressure to maintain an ideal online presence. These factors may, in turn, be associated with greater cognitive and emotional demands and lower engagement in academic activities, although these mechanisms were not directly examined in the present study.

These findings may be particularly relevant within collectivist social contexts, where maintaining interpersonal connectedness and social interaction are culturally emphasized. In such settings, socially motivated engagement with social media may be more time-consuming and emotionally involving and may therefore be associated with poorer academic performance. Collectively, these findings suggest that entertainment- and socialization-oriented social media use are associated with poorer academic performance among medical students.

Our findings differ from previous studies regarding the association between informational motives and academic performance (66, 83, 84). However, the present findings are consistent with those of Cuong et al. (62), as informational motives showed no significant relationship with GPA. This suggests that using social media for general information-seeking does not necessarily translate into improved academic performance. One possible explanation relates to the nature of informational engagement on social media platforms. Information obtained through social media is typically broad, unstructured, and insufficiently aligned with formal academic curricula or assessment requirements. Consequently, although students may frequently engage in information-seeking behaviors, such activities do not necessarily support structured learning, examination preparation, or deep conceptual understanding. In addition, informational use may be associated with cognitive overload due to exposure to large volumes of diverse and unfiltered content. This may be associated with reduced attention span and make it difficult for students to identify and focus on academically relevant material, thereby limiting potential learning benefits (85). Furthermore, informational motives are inherently heterogeneous in nature, ranging from general news consumption to academic-related searching. Such variability may partly explain the absence of a consistent association with GPA, particularly when only a limited proportion of informational use is directly related to coursework. Overall, these results suggest that informational motives alone may be insufficient to be associated with academic performance unless the information accessed is clearly academically relevant in nature and effectively integrated into structured learning activities.

The findings further demonstrated that social media use motives were differentially associated with dimensions of perceived stress, including perceived helplessness and lack of self-efficacy, thereby supporting H2. Consistent with Uses and Gratifications Theory, these findings suggest that the psychological association of social media depend not only on the extent of use but also on the underlying motivations driving engagement.

Academic motives demonstrated significant negative associations with both perceived helplessness and lack of self-efficacy, indicating that students who primarily use social media for educational purposes tend to experience lower stress levels and greater confidence in managing academic demands. These findings are consistent with previous studies suggesting that academically oriented social media use may facilitate collaborative learning, improve access to educational resources, and strengthen academic engagement (6, 11). One plausible explanation is that academically motivated social media use may be linked to a greater sense of academic preparedness, competence, and control over learning tasks. Students may use social media for academic purposes to discuss coursework, exchange educational materials, access lecture-related information, and participate in peer-supported learning (6, 86), all of which may be associated with lower levels of uncertainty and more effective coping with academic demands. Such structured and goal-directed engagement may also be associated with greater perceived ability to effectively manage academic challenges, thereby being associated with lower feelings of helplessness and low self-efficacy. In highly demanding medical education settings, academically oriented social media use may therefore function as a supportive educational resource rather than be associated with a psychological burden.

In the unadjusted analyses, entertainment motives were positively associated with both perceived helplessness and lack of self-efficacy, indicating that students who primarily used social media for entertainment initially reported higher levels of perceived stress. One possible explanation is that entertainment-oriented social media use may be associated with prolonged leisure engagement, emotional escapism, or reduced focus on academic activities, which may in turn be associated with greater academic pressure and lower confidence in coping with academic demands. Previous research has likewise suggested that entertainment-oriented social media use is associated with maladaptive coping, excessive engagement, and poorer psychological well-being (36, 87). However, these associations were no longer statistically significant after adjustment for demographic characteristics, social media use intensity, and the remaining social media use motives. This finding suggests that entertainment-oriented social media use does not independently contribute to perceived stress once overlapping motivational dimensions are taken into account. Instead, its apparent relationship with stress may largely reflect variance shared with other social media use motives.

Similarly, socialization motives demonstrated significant positive associations with perceived helplessness and lack of self-efficacy. These findings align with previous studies reporting that socially driven social media use is associated with emotional strain, social comparison, fear of missing out, and psychological distress (88, 89). One possible explanation is that students who use social media primarily for social interaction may experience continuous pressure to remain socially connected and responsive in online environments. Maintaining online communication and managing digital social relationships often involves frequent checking behaviors, prolonged interaction, and constant monitoring of social feedback, all of which may contribute to attentional fragmentation and emotional exhaustion. In addition, socialization-oriented engagement may be associated with greater exposure to upward social comparison and greater concerns regarding online self-presentation, which have been associated with higher levels of psychological pressure and lower perceived ability to cope effectively with academic demands. These findings may be particularly relevant within collectivist social contexts such as Saudi Arabia, where interpersonal relationships and social connectedness are culturally emphasized. In such environments, socially motivated social media engagement may be more emotionally involving and psychologically demanding, which may help explain its observed association with higher levels of perceived stress. Importantly, unlike entertainment motives, these associations remained statistically significant even after adjustment for demographic characteristics, social media use intensity, and the remaining social media use motives, indicating that socialization motives remained independently associated with both perceived helplessness and lack of self-efficacy.

In contrast, informational motives did not demonstrate significant associations with either perceived helplessness or lack of self-efficacy. These findings partially diverge from previous studies reporting associations between information-related social media use and psychological distress or information overload (25, 85). One possible explanation is that informational engagement on social media represents a relatively neutral and heterogeneous form of use that may produce both beneficial and adverse psychological effects depending on the nature of the information being consumed. While access to useful and relevant information may occasionally support coping and academic functioning, excessive exposure to fragmented, unstructured, or non-academic information may also contribute to cognitive overload without necessarily influencing overall stress levels. Therefore, the absence of a significant relationship in the present study may reflect the diverse and inconsistent nature of informational social media engagement across students.

The findings further demonstrated that perceived stress was significantly associated with academic performance among Saudi medical students, thereby supporting H3. Both perceived helplessness and lack of self-efficacy showed consistent negative associations with GPA across adjusted and unadjusted models, indicating that students experiencing higher levels of stress tended to report poorer academic performance. These findings are consistent with previous research showing that elevated stress adversely affects academic functioning, concentration, motivation, and educational achievement among university and medical students (34, 50, 90–92). One possible explanation is that high levels of perceived stress may be associated with poorer cognitive and emotional functioning, thereby potentially limiting students ability to effectively manage academic demands. Medical education is characterized by intensive coursework, frequent examinations, and persistent academic pressure, all of which require strong concentration, self-regulation, and psychological resilience. Students experiencing greater perceived helplessness may feel overwhelmed by academic responsibilities and perceive limited control over their ability to cope with educational challenges. Such perceptions may be associated with lower motivation, poorer decision-making, and greater susceptibility to academic exhaustion and procrastination, all of which have been associated with poorer academic achievement. Similarly, lack of self-efficacy demonstrated a significant negative relationship with GPA, suggesting that students who lack confidence in their ability to cope with stress and academic demands tend to perform more poorly academically. This finding aligns with previous literature emphasizing the critical role of self-efficacy in academic adjustment and educational performance (93, 94). Students with lower stress-related self-efficacy may encounter greater difficulty organizing study activities, maintaining motivation, and persisting through academic challenges, thereby being associated with lower academic productivity and performance. The present findings are also consistent with the Transactional Model of Stress and Coping, which proposes that stress emerges when individuals perceive environmental demands as exceeding their available coping resources (45). In the context of medical education, prolonged exposure to academic stress may be associated with poor memory, concentration, emotional regulation, and learning efficiency- all of which are essential for academic success. Persistent stress may additionally be linked with maladaptive coping behaviors such as procrastination, avoidance, emotional withdrawal, and poorer academic engagement, further being associated with poorer academic functioning. These findings may be particularly important within medical education settings, where students are exposed to continuous academic pressure and high performance expectations. In such environments, unmanaged stress may accumulate over time and substantially linked with both psychological well-being and educational achievement. Collectively, the findings suggest that perceived stress represents a significant psychological factor associated with academic performance and underscore the importance of stress management and psychological support interventions in improving academic functioning among medical students.

The mediation analyses further demonstrated that perceived stress significantly mediated the relationship between selected social media use motives and academic performance among Saudi medical students, thereby partially supporting H4. Specifically, both perceived helplessness and lack of self-efficacy emerged as significant psychological pathways linking academic and socialization motives with GPA. These findings suggest that the observed associations between social media use motives and academic performance may occur not only directly but also indirectly through students’ stress experiences, consistent with Uses and Gratifications Theory and stress-based frameworks (44, 45). However, given the cross-sectional design, these indirect associations should not be interpreted as evidence of causal mediation.

Academic motives demonstrated significant positive indirect effects on GPA through lower perceived helplessness and lower lack of self-efficacy. Students using social media for academic purposes reported lower stress levels, which in turn were associated with better academic performance. One possible explanation is that academically motivated social media use facilitates access to educational resources, peer collaboration, and academic support, thereby strengthening students’ confidence in managing academic demands. These findings align with previous literature showing that academically oriented social media use may enhance academic engagement and learning adjustment (6, 11, 21). In contrast, socialization motives demonstrated significant negative indirect effects on GPA through perceived helplessness and lack of self-efficacy. Students who primarily used social media for social interaction reported higher levels of stress, which in turn was associated with lower GPA. Frequent online interaction may be associated with greater emotional exhaustion, social comparison, fear of missing out, and attentional fragmentation, all of which may interfere with academic focus and coping capacity (37, 89, 95–97). These effects may be particularly relevant in collectivist social contexts where interpersonal connectedness is strongly emphasized.

In the unadjusted analyses, entertainment motives demonstrated significant negative indirect effects on GPA through both perceived helplessness and lack of self-efficacy, suggesting that students who primarily used social media for entertainment reported higher stress levels, which in turn were associated with poorer academic performance. However, these indirect effects were no longer statistically significant after adjustment for demographic characteristics, social media use intensity, and the remaining social media use motives. This finding suggests that the significant mediation observed in the unadjusted analyses may have been attributable largely explained by variance shared with other social media use motives, particularly socialization motives, rather than reflecting an independent stress-mediated pathway. Nevertheless, entertainment motives continued to demonstrate significant direct negative associations with GPA after adjustment, indicating that their relationship with academic performance is likely explained through mechanisms other than perceived stress. Previous research has suggested that entertainment-oriented social media use may be associated with poorer academic performance through factors such as greater distractibility, higher levels of procrastination, reduced study time, and poorer self-regulation (36, 87, 98). These mechanisms were not directly assessed in the present study and therefore warrant further investigation. The disappearance of the indirect effects after mutual adjustment may suggest that the previously observed mediation pathways were largely attributable to variance shared across multiple social media use motives rather than to entertainment motives independently. Because students frequently engage with social media for several purposes simultaneously, entertainment-oriented use overlaps considerably with other forms of engagement, particularly socialization. Once this shared variance was statistically controlled, entertainment motives no longer demonstrated unique associations with perceived helplessness or lack of self-efficacy. This finding highlights the importance of mutually adjusting correlated motivational dimensions when examining the psychological pathways linking social media use motives with academic outcomes. More broadly, these findings demonstrate the methodological value of simultaneously adjusting for conceptually related social media use motives in mediation analyses. Because students often use social media for multiple purposes, the motivational dimensions are inherently interrelated and may share substantial variance. Estimating all relevant motive dimensions within the same adjusted model allows the unique association of each motive with perceived stress and academic performance to be evaluated while accounting for this shared variance. Consequently, the adjusted models provide a more conservative and theoretically informative assessment of the distinct relationships associated with each social media use motive, reducing the likelihood that observed indirect associations simply reflect overlap with other correlated motivations. This methodological approach may be particularly valuable for future studies examining multiple, conceptually related predictors within mediation frameworks.

Similarly, informational motives did not demonstrate significant indirect effects through either stress dimension. This finding suggests that informational social media use may not be consistently associated with academic performance through stress-related pathways. One possible explanation is that informational engagement includes both academically relevant and non-academic information-seeking behaviors, resulting in inconsistent psychological and academic effects (25, 85). Overall, the findings highlight perceived stress as an important psychological mechanism associated with the relationship between social media use motives with academic performance. Academically oriented motives may be associated with better academic performance through lower levels of perceived stress and greater academic confidence, whereas socialization motives may be associated with poorer academic performance alongside higher perceived helplessness and lower self-efficacy.

In contrast, although entertainment motives remained directly associated with poorer academic performance, their indirect effects through perceived stress were no longer statistically significant after adjustment for demographic characteristics and the remaining motive dimensions, suggesting that alternative psychological or behavioral mechanisms may better explain this relationship.

### Limitations and future directions

Several limitations should be considered when interpreting the findings of the present study. First, the cross-sectional design limits the ability to establish causal relationships between social media use motives, perceived stress, and academic performance. Although the mediation analyses provide evidence for indirect associations, causal direction cannot be definitively determined. Future longitudinal and experimental studies are needed to establish temporal ordering and evaluate the directionality of these relationships. Longitudinal studies may also help clarify the temporal relationships among social media use motives, actual usage behaviors, perceived stress, and academic performance, and provide greater insight into how these factors interact over time. Second, the study relied entirely on self-reported measures, including GPA, social media use motives, and perceived stress, which may increase the risk of recall bias, social desirability bias, and common method variance. In particular, self-reported academic performance may not fully reflect objective academic achievement. Furthermore, academic performance was assessed using self-reported GPA categories rather than exact GPA values or official academic records. Although this approach was adopted to preserve participant confidentiality, categorical GPA measures provide less precision than objective continuous indicators of academic achievement and may not fully satisfy the assumptions underlying parametric analyses. Consequently, caution is warranted when interpreting the magnitude of the observed associations involving academic performance. Nevertheless, the sensitivity analyses demonstrated highly consistent findings using both the continuous and original ordinal operationalization’s of GPA, supporting the robustness of the observed results. However, self-reported GPA remains susceptible to recall inaccuracies and social desirability bias, and some degree of measurement error cannot be ruled out. Future studies should consider verifying academic performance using official institutional academic records or other objective indicators whenever feasible to improve measurement precision. Additionally, although Harman’s single-factor test indicated that common method variance was unlikely to pose a substantial threat, the possibility of method-related bias cannot be completely ruled out. All major study variables, including social media use motives, perceived stress, and academic performance, were assessed using self-reported measures collected from the same participants at a single time point. Consequently, shared method variance may have contributed to the observed associations. Future studies should incorporate objective measures of social media engagement, such as screen-time records, platform usage logs, digital trace data, and official academic records, to complement self-reported data and enhance measurement accuracy. Third, the study employed a convenience sample drawn from a single university, which may limit the external validity and generalizability of the findings. Although the student population included individuals from different regions of Saudi Arabia, the sample cannot be considered nationally representative of Saudi medical students. Consequently, caution is warranted when generalizing the findings to students from other institutions, regions, academic disciplines, cultural contexts, or educational systems. Furthermore, medical students experience unique academic pressures and patterns of social media engagement that may differ from those of the general university population. Future multi-institutional studies using probability-based sampling methods are needed to enhance the generalizability of the findings. Fourth, although the study examined multiple social media use motives, social media behavior remains highly complex and multidimensional. The present study did not assess detailed patterns of social media engagement, including platform-specific use, frequency of use, active versus passive engagement, time spent on specific activities, content types, or problematic social media behaviors. These factors may differentially relate to perceived stress and academic performance and could provide additional insight into the mechanisms underlying the observed associations. Future research should incorporate more comprehensive assessments of social media behavior to better understand how specific patterns of engagement relate to students’ psychological well-being and academic outcomes. Fifth, an important distinction exists between reported social media use motives and actual social media use behaviors. Although the study assessed participants’ self-reported motives for using social media, these motives may not necessarily correspond to how students actually allocate their time or engage with specific online activities. For example, students reporting academic motives may still spend considerable time consuming entertainment-oriented content, whereas those endorsing socialization motives may also engage in academically relevant activities. Consequently, the observed relationships may reflect a combination of motivational orientations and actual usage behaviors that could not be disentangled within the present study. Future research should incorporate objective behavioral indicators, platform-specific activity measures, or digital trace data to examine the extent to which reported motives correspond to actual social media behaviors and to better differentiate the effects of motivations versus behaviors on stress and academic performance. Sixth, the social media use motives examined in this study are not mutually exclusive, and students may simultaneously engage with social media for academic, socialization, entertainment, and informational purposes. Although the present study adopted a variable-centered approach by examining each motive as a separate predictor, this approach does not capture the possibility that multiple motives may coexist within individuals in distinct patterns. Consequently, the observed associations may not fully reflect the complexity of motive combinations underlying social media engagement. Future research may benefit from employing person-centered approaches, such as Latent Profile Analysis or Latent Class Analysis, to identify distinct social media user profiles and examine how different configurations of motives relate to perceived stress and academic performance. Future studies would also benefit from incorporating objective behavioral indicators, including smartphone screen-time records, digital trace data, learning management system (LMS) activity logs, and ecological momentary assessment (EMA), to capture real-time patterns of social media engagement and academic behavior. Combining these approaches with person-centered analytical methods such as Latent Profile Analysis may provide a more comprehensive understanding of naturally occurring social media use profiles and their psychological and academic correlates. Seventh, although the adjusted mediation models controlled for demographic characteristics, overall social media use, and the remaining social media use motive dimensions, residual confounding cannot be completely ruled out. Several potentially important variables were not assessed, including sleep quality, personality traits (e.g., conscientiousness), self-regulation, attention-related difficulties, mental health status (e.g., anxiety and depressive symptoms), study habits, academic motivation, and social support. These factors may be associated with social media use motives, perceived stress, and academic performance and therefore may have contributed to the observed associations. Finally, although the mediation models explained a substantial proportion of the variance in academic performance, GPA is associated with a broad range of academic, psychological, behavioral, and environmental factors beyond those included in the present study. Accordingly, the findings should be interpreted as reflecting specific theory-driven associations rather than a comprehensive explanation of academic performance among medical students. Future longitudinal studies incorporating these variables would provide a more comprehensive understanding of the independent relationships among social media use motives, perceived stress, and academic performance.

### Theoretical and practical implications

The present study offers several important theoretical contributions to the growing literature on social media use, psychological well-being, and academic functioning among university students. First, the findings extend the application of Uses and Gratifications Theory by demonstrating that the associations of social media are not determined solely by frequency or duration of use, but rather by the underlying motives driving engagement. By differentiating between academic, entertainment, informational, and socialization motives, the study highlights the multidimensional nature of social media use and provides evidence that different motives are associated with distinct academic and psychological outcomes. These findings contribute to resolving inconsistencies in previous literature that often conceptualized social media use as a single, uniform construct. Second, the findings provide additional support for the Transactional model of Stress and Coping by demonstrating that social media use motives are associated with academic performance partly through stress-related psychological processes. Academically oriented social media engagement was associated with lower levels of perceived helplessness and lack of self-efficacy, whereas socialization-driven engagement was consistently associated with elevated stress perceptions. Although entertainment-oriented engagement remained negatively associated with academic performance, its association with perceived stress was no longer statistically significant after adjustment for demographic characteristics, social media use intensity, and the remaining social media use motives. These findings suggest that different forms of social media engagement may be associated with either more adaptive or less adaptive coping capacities depending on the motivations underlying use. By integrating the Transactional Model of Stress and Coping with Uses and Gratifications Theory, the study provides a more comprehensive framework for understanding the associations among motive-specific social media engagement, perceived stress, and academic performance. Third, the study advances existing literature by identifying perceived stress, operationalized through perceived helplessness and lack of self-efficacy, as an important psychological mechanism underlying the relationship between social media use motives and academic performance. The findings demonstrate that social media motives are associated with academic outcomes both directly and indirectly through stress-related pathways. This contributes to the limited body of research examining mediating psychological processes linking social media behavior with educational outcomes, particularly among medical students within the Saudi and Gulf context.

From a practical perspective, the findings highlight the importance of promoting academically oriented and purposeful social media engagement among medical students. Universities and medical colleges may benefit from integrating structured educational use of social media into learning environments through academic discussion groups, peer-learning platforms, and digital resource-sharing initiatives. Encouraging productive and goal-directed online engagement may help improve academic performance while reducing stress-related academic difficulties. The findings also emphasize the need for psychological support and stress-management interventions within medical education settings. Since socialization-driven social media use remained independently associated with higher perceived stress, and both entertainment- and socialization-driven social media use were associated with poorer academic performance, educational institutions should provide awareness programs focusing on healthy digital habits, self-regulated learning, time management, and balanced social media use. Workshops addressing emotional regulation, coping strategies, and digital well-being may help promote healthier online engagement and support better academic functioning among students. In addition, the results may inform policymakers and academic advisors regarding the development of student support programs targeting digital behavior and psychological well-being. Given the exceptionally high prevalence of social media use among Saudi university students, interventions aimed at improving responsible and academically productive social media engagement may have meaningful educational and mental health benefits. Overall, the study underscores the importance of considering not only how much students use social media, but also why they use it, when evaluating its impact on academic and psychological outcomes.

## Conclusion

The present study demonstrated that social media use motives are differentially associated with academic performance and perceived stress among Saudi medical students. Academically oriented social media use was associated with better academic performance and lower levels of perceived stress, whereas socialization motives were associated with poorer academic performance, higher perceived helplessness, and lower self-efficacy. Entertainment motives remained negatively associated with academic performance but were no longer independently associated with perceived stress after adjustment for demographic characteristics, social media use intensity, and the remaining social media use motives. In contrast, informational motives showed no significant relationship with either academic performance or perceived stress. The findings further revealed that perceived stress, particularly perceived helplessness and lack of self-efficacy, was negatively associated with academic performance and significantly mediated the relationship between selected social media use motives and GPA. However, because of the cross-sectional design, these mediation findings should be interpreted as evidence of indirect statistical associations rather than causal pathways. These results suggest that the observed associations between social media use motives and academic performance may occur both directly and indirectly through stress-related psychological mechanisms. Overall, the study highlights that the observed relationships between social media use and academic functioning appear to depend largely on why students use social media rather than on social media use per se. Academically purposeful engagement was associated with better academic-related outcomes and lower stress, whereas socialization-driven use was consistently associated with higher stress and poorer academic performance. Although entertainment-oriented use remained negatively associated with academic performance, its relationship with perceived stress was no longer statistically significant after adjustment. The findings underscore the importance of promoting healthy and academically productive social media engagement, alongside stress-management and digital well-being interventions, within medical education settings.

## Data Availability

The raw data supporting the conclusions of this article will be made available by the authors, without undue reservation.
