# Correction: Social media use motives and academic performance among Saudi medical students: the mediating role of perceived stress

**DOI:** 10.3389/fmed.2026.1970403

**Published:** 2026-08-28

**Authors:** Ayoob Lone, Imtiyaz Ahmad Dar, Mohammed Farhan AlFarhan, Syed Gulfishan, Abdul Wahab Pathath, Sayed Abdul Qadar Quadri, Nawaf Al Khashram, Abdullah Abdulaziz Alnaim, Maryam Jan

**Affiliations:** 1 Department of Clinical Neurosciences, College of Medicine, King Faisal University, Al Ahsa, Saudi Arabia; 2 Department of Psychology, University of Kashmir, Srinagar, Jammu and Kashmir, India; 3 Department of Surgery, College of Medicine, King Faisal University, Al Ahsa, Saudi Arabia; 4 Department of Biomedical Sciences, College of Medicine, King Faisal University, Al Ahsa, Saudi Arabia; 5 Medical Education Department, College of Medicine, King Faisal University, Al Ahsa, Saudi Arabia; 6 Department of Family and Community Medicine, College of Medicine, King Faisal University, Al Ahsa, Saudi Arabia; 7 Department of School Education, Srinagar, Jammu and Kashmir, India

**Keywords:** academic performance, medical students, perceived stress, Saudi Arabia, social media motives

An incorrect grant number was provided for the Deanship of Scientific Research and the Vice Presidency for Graduate Studies and Scientific Research, King Faisal University. The correct number is “Grant No. KFU263679”.

The original version of this article has been updated.

